# Gut microbiota and oleoylethanolamide in the regulation of intestinal homeostasis

**DOI:** 10.3389/fendo.2023.1135157

**Published:** 2023-04-05

**Authors:** Carlotta De Filippo, Alessia Costa, Maria Vittoria Becagli, Mariela Mejia Monroy, Gustavo Provensi, Maria Beatrice Passani

**Affiliations:** ^1^ Istituto di Biologia e Biotecnologia Agraria, Consiglio Nazionale delle Ricerche, Pisa, Italy; ^2^ Dipartimento di Scienze della Salute, Università di Firenze, Firenze, Italy; ^3^ Dipartimento di Neurofarba, Università di Firenze, Firenze, Italy

**Keywords:** *dysbiosis*, inflammation, obesity, gut barrier permeability, intestinal physiology, metabolic diseases

## Abstract

A vast literature strongly suggests that the endocannabinoid (eCB) system and related bioactive lipids (the paracannabinoid system) contribute to numerous physiological processes and are involved in pathological conditions such as obesity, type 2 diabetes, and intestinal inflammation. The gut paracannabinoid system exerts a prominent role in gut physiology as it affects motility, permeability, and inflammatory responses. Another important player in the regulation of host metabolism is the intestinal microbiota, as microorganisms are indispensable to protect the intestine against exogenous pathogens and potentially harmful resident microorganisms. In turn, the composition of the microbiota is regulated by intestinal immune responses. The intestinal microbial community plays a fundamental role in the development of the innate immune system and is essential in shaping adaptive immunity. The active interplay between microbiota and paracannabinoids is beginning to appear as potent regulatory system of the gastrointestinal homeostasis. In this context, oleoylethanolamide (OEA), a key component of the physiological systems involved in the regulation of dietary fat consumption, energy homeostasis, intestinal motility, and a key factor in modulating eating behavior, is a less studied lipid mediator. In the small intestine namely duodenum and jejunum, levels of OEA change according to the nutrient status as they decrease during food deprivation and increase upon refeeding. Recently, we and others showed that OEA treatment in rodents protects against inflammatory events and changes the intestinal microbiota composition. In this review, we briefly define the role of OEA and of the gut microbiota in intestinal homeostasis and recapitulate recent findings suggesting an interplay between OEA and the intestinal microorganisms.

## 1 Synthesis and degradation of OEA

The oleic acid derivative OEA belongs to the so-called endocannabinoid-like compounds as it shares with anandamide a similar chemical structure and the enzymes for the biosynthesis and degradation, although it does not bind to either CB_1_ or CB_2_ receptors. Hence, it is more appropriate to assign OEA, along with palmitoylethanolamide (PEA) and similar bioactive lipids to the endocannainoidome ensemble. As other *N*-acylethanolamines (NAEs), OEA is found in various tissues and its synthesis increases or decreases according to the different homeostatic functions that OEA controls. OEA endogenous levels depend on the balance between biosynthesis and deactivation processes; it is involved in the regulation of lipid metabolism, body weight and feeding behavior (1–5).

Presumably, fat digestion in the small intestine triggers the release of free oleic acid, which is internalized by the enterocytes lining the lumen of the proximal gut and is directed to produce either chylomicrons or OEA. Indeed, duodenal infusion of individual nutrients revealed that fat, in particular oleic acid, is a potent stimulator of OEA synthesis, whereas proteins and sugar are not (6, 7). OEA is synthesized *via* a two-step reaction mechanism catalyzed by the sequential action of N-acyl transferase (NAT) (8–10) and N-acyl-phosphatidylethanolamine-selective phospholipase D (NAPE-PLD) (11–14). NAPE-PLD is widely expressed in animal tissues, including various regions of the rat brain (15), and in the enterocytes of the mouse duodenum, where its activity and expression are enhanced by feeding (16). OEA synthesis is also enabled by an alternative biosynthetic pathway *via* α/β-hydrolase-4 (ABHD4) and glycerophosphodiesterase1 (GDE1) (17, 18).

OEA released from small-intestinal enterocytes of various species indirectly signals satiety to hypothalamic nuclei, in particular to histaminergic neurons in the tuberomamillary nucleus. Indeed, we demonstrated that OEA requires a functioning brain histaminergic system to fully exert its satiating effect (19).

Newly formed OEA binds to peroxisome proliferator-activated receptors-α (PPAR-α), which activate sensory fibres of the vagus nerve through an ill-defined mechanism, promoting satiety (4). OEA signaling takes place also *via* the transient receptor potential vanilloid 1 (TRPV1), which presumably mediates intestinal hyperpermeability (20) and visceral pain (21).

Other key elements that bind OEA are the membrane glycoprotein fatty-acid transporter CD36 which plays an obligatory role in food-stimulated OEA production, and the Gαs-coupled receptor GPR119. CD36 binds long-chain fatty acids and translocate them through cell membranes (22); presumably it acts as a biosensor for food derived oleic acid, as its deletion abrogates food-stimulated production of OEA (23). The GPR119 activation is thought to mediate OEA regulation of glucose homeostasis [reviewed in (24)].

OEA is hydrolyzed into oleic acid and ethanolamine, the primary mechanism through which its biological actions are terminated. The structurally unrelated enzymes involved in this transformation are the fatty acid amide hydrolase (FAAH) and N-acylethanolamine acid amidase (NAAA). FAAH is highly expresses in the central nervous system (CNS), liver and small intestine (25), whereas NAAH is distributed in the liver, brain, kidney (26) as well as well as in epithelial and lamina propria cells of the mouse jejunum (16).

## 2 Dietary regulation of OEA production in different organs and tissues

### 2.1 Regulation of OEA levels in the intestine

Not only food composition is a key element that regulates the complex processes of OEA metabolism, but also its availability; animal studies have shown that food deprivation for 24h decreases OEA biosynthesis in the mucosal layer of rat duodenum and jejunum, whereas OEA levels increase upon refeeding (16). On the other hand, excessive high-fat exposure suppresses intestinal OEA synthesis and renders the homeostatic processes controlled by this NAE dysfunctional (27). This suggests that a diet too rich in fat promotes overeating, at least in part, by suppressing the satiating effects of gut derived OEA.

Overconsumption of dietary fats also dampens the activity of a brain reward circuit involving dopamine release, which leads to compensatory intake of even more high-fat foods to restore reward sensitivity (28). An elegant work by (27) showed that infusion of OEA *via* intraperitoneal catheters to mice that had been accustomed to a high-fat diet, restored the brain dopaminergic response, and these animals began to eat more low-fat foods. In this regard, another study demonstrated that in a rat model of bariatric surgery, the ultimate treatment strategy for long-lasting weight loss in patients with morbid obesity, ingested fat mobilizes OEA production, which is associated with vagus nerve-dependent increase in dopamine D1 receptor expression and striatal dopamine release (29).

Furthermore, a high fat-high sucrose (HFHSD) diet fed to male mice induced early and persistent weight gain, hyperinsulinemia and glucose intolerance, along with alteration in the endocannabinoidome. In particular, the HFHS diet elevated AEA levels, decreased OEA and PEA in the plasma and changed, in a segment-specific fashion, the relative abundance of several intestinal microbiota genera (30). Hence, the authors demonstrate the existence of an interaction between the endocannabinoidome and intestinal microbiota during a maladaptive response that leads to diet-induced obesity and metabolic complications.

All these observations indicate that the intestinal regulation of OEA production may have fundamental consequences on eating misbehavior of human patients. Indeed, the clinical implications of these findings are beginning to emerge; for instance, a couple of studies demonstrated the beneficial effects of OEA in morbid obese patients (31) and in obese patients diagnosed with non-alcoholic fatty liver disease (NAFLD) (32). Furthermore, animal data indicate a cholesterol lowering effect of OEA treatment (33) and a clinical investigation showed a positive correlation between serum OEA levels and high-density lipoproteins (HDL), and a negative correlation with BMI and anthropometric measurements in hemodialysis patients (34). However, discordant results were obtained in subjects fed a high-protein diet, as OEA was found to be positively associated with cardiometabolic risk markers, such as total and LDL serum cholesterol (35). As suggested by the authors, establishing whether OEA provides a compensatory/regulatory factor in these human experimental settings require further research.

### 2.2 Regulation of OEA content in the liver and fat mass

An interesting aspect of OEA metabolism is that its levels change according to the nutrient status with different modalities in different tissues and organs; for instance, contrary to what happens in the intestine, fasting *increases* the content of OEA in the white adipose tissue and liver and return to basal levels upon refeeding (16, 36). The differential regulation of OEA biosynthesis substantiates its regulatory role in distinct aspects of energy balance, not only on energy intake (1, 37), but also in fat utilization (38) and ketone bodies synthesis (39). This regulation is relevant for the synthesis of hepatic ketone bodies as source of energy during food scarcity. Ketogenesis is a crucial metabolic response to prolonged periods of food paucity and is initiated by the stimulation of PPAR-α which control transcription of many genes involved in ketogenesis and fatty acid oxidation in response to fasting (40).

The newly formed OEA during fasting together with lipolysis-derived free fatty acids activate PPAR-α in the hepatic tissue. This mechanism is presumably based on extrahepatic mast cells secretion of histamine into the portal circulation. We recently demonstrated that histamine acts as a paracrine signaling system which enhances ketogenesis during fasting, as demonstrated with pharmacological and genetic treatments which inactivate the histaminergic signaling and consequently diminish both ketogenesis and hepatic OEA synthesis (39). Long exposure to a high fat diet disrupts this homeostatic process as it suppresses both fasting-dependent histamine release in the portal blood and OEA production in the liver. When OEA is administered exogenously, though, all fat-induced markers of liver steatosis such as fibrosis, lipid accumulation and several parameters associated with oxidative stress, are reduced (41, 42). Furthermore, OEA differently regulates the expression of nuclear factor erythroid-derived 2-related factor 1 (Nrf1) and Nrf2, two transcription factors involved in the control of lipid metabolism and antioxidant genes (41) and reduces inflammation and fat accumulation in a rat model of non-alcoholic fatty liver (NAFLD) (43). These fundamental results suggest new targets of the protective effect of OEA in the liver.

In white adipose tissue, β-adrenergic receptor activation and cold exposure stimulate OEA production (44), which is responsible for stimulating glycerol and fatty acid release, and lipolysis (45). Short-term cold exposure and acute β3-adrenoceptor activation elevate OEA levels also in the brown adipose tissue, suggesting a role for this NAE in the control of thermogenesis (17).

OEA regulates lipid transport into the adipose tissue of High Fat Diet-fed mice, contributing to lower adiposity (46), and controls lipid metabolism in genetically obese rats and Diet-Induced Obesity (DIO) mice (33, 38). In a clinical context, it is interesting that OEA contributes to the reduction of inflammation associated with gastric bypass surgery of morbidly obese subjects (31). These authors designed a translational project including clinical and *in vitro* studies with morbid obese patients submitted to gastric bypass surgery (GBS) and found an inverse correlation independent of body mass index between palmitoylethanolamide (PEA) and OEA levels, and inflammatory molecules in the adipose tissue.

The potential clinical utility of OEA in the treatment of obesity has been addressed in clinical and preclinical studies with overall positive results. As examples, the administration of OEA in synergy with a β-adrenergic receptor agonists caused a significant fat mass reduction and enhanced energy expenditure in rats, along with decreased plasma levels of leptin and TNFα (47). More recent clinical trials in obese subjects and patients with NAFLD demonstrated that OEA supplementation decreased anthropometric measures including body mass index and waist circumference (32, 48).

## 3 OEA is a predominant player in intestinal physiology

Many bioactive lipids regulate several physiological processes that maintain the gut-barrier integrity, control inflammation, pain and energy metabolism (see (49) for a comprehensive review). OEA in particular exerts prominent roles in intestinal physiology; it reduces intestinal motility together with other lipid mediators such as PEA and oleamide, suggesting a potential target for the development of efficient drugs to reduce intestinal motility (50); OEA presumably partakes in the maintenance of normal glucose homeostasis as it increases the secretion of Glucagon-like Peptide-1 (GLP-1, an intestinal hormone with potent insulinotropic effects) by binding to GPR119 expressed on enteroendocrine L-cells (51). Experiments *in vitro* demonstrated that OEA decreased intestinal epithelial cells permeability (20), and we recently reported that OEA affects the polarization of T_H_ lymphocytes in intestinal Peyer’s patches (52). Peyer’s patches, together with small intestine epithelial cells, are in the optimal position to discriminate between commensal bacteria and pathogens. Several cytokines and chemokines released by lymphocytes are modulated by OEA, as it decreases release of proinflammatory IFNγ, IL6, IL17, IL4, and chemokines CXCL1 and CXCL2, which are necessary to recruit neutrophils in inflammation. Immunohistochemical studies demonstrated that the expression of PPAR-α, for which OEA has a high affinity, was decreased in biopsies of the colonic epithelium taken from patients with active colitis compared to healthy subjects (53). We therefore evaluated the protective effects of OEA administration in a mouse model of ulcerative colitis, by exposing mice to dextran sodium sulphate (DSS). It was found that a sub-chronic treatment with OEA ameliorated the inflammatory profile of DSS-treated mice by decreasing systemic and colonic expression of pro-inflammatory cytokines as well as the expression of inflammatory cytokines in mesenteric lymph nodes of diseased mice (54). Furthermore, OEA exerted a protective action on the gut barrier by restoring mRNA transcription of tight junctions and other factors such as mucin that maintain colon integrity (54). Altogether these findings are of relevance in the context of intestinal pathologies and prompted the question of whether OEA and the intestinal microbiota somehow cooperate to maintain the gastrointestinal functional integrity and physiology.

## 4 Intestinal microbiota: A real metabolizing organ

### 4.1 Diet composition affects the microbiota profile

The intestinal microbiota, a complex microbial community residing in the gastrointestinal tract, has a major role in maintaining the health of the host organism, providing essential metabolic capabilities, such as the availability of nutrients, vitamins, energy, as well as contributing to the detoxification and resistance towards infectious diseases (55). The intestinal microbiota is also capable of metabolizing biologically active molecules from food, which would otherwise be discarded from the intestinal tract, recovering energy, producing “microbiota-derived metabolites” that orchestrate and support physiological responses in the host, including metabolism, immune response, inflammation, and defense against infections (56). The intestinal microbiota is also capable of influencing the host energy balance, as demonstrated by several studies on germ-free animals. These require 30% more energy in the normal diet to maintain the ideal weight (57). Indeed, intestinal bacteria draw the necessary energy from sugars and proteins metabolism, through the process of fermentation. The transformation of non-digestible polysaccharides of the diet (cellulose, hemicellulose, pectin, non-digestible starch) takes place thanks to bacterial enzymes, such as glycoside hydrolase which converts glycans into useable sugars (>81 different glycoside hydrolase families) and transform food-derived components into volatile substances (carbon dioxide, hydrogen sulphide) and short-chain fatty acids (SCFAs) such as acetic, butyric and propionic acid, derived from the fermentation of the fibers which represent the main source of nourishment of the colonic mucosa (58). The composition of the human intestinal microbiota is extremely variable between healthy people as well as between individuals with different BMI (lean and obese) (59). The microbiota is very sensitive to variations in the diet, producing relevant changes in host metabolism such as absorption, storage, and metabolism of dietary lipids, which are tightly regulated by the intestinal microbiota. Understanding the interactions between diet and intestinal microbiota is a topic of great interest to cure and prevent many diseases when gut microorganisms seem to be involved. The elegant work by David and collaborators (60) carried out on ten volunteers who agreed to follow a strictly vegetarian diet for 5 days and then switched to a strictly carnivorous diet in the following 5 days, demonstrated that the intestinal microbial communities react very quickly. Within 24-48 hours the composition of the intestinal microbiota changed significantly: during the vegetarian diet, bacterial species digesting complex carbohydrates prevailed, whereas during the animal proteins-based diet, *Bilophila wadsworthia* was selected, a bacterial species that metabolizes proteins and toxic compounds derived from the combustion of meat, with a strong proinflammatory potential.

### 4.2 Intestinal homeostasis and short chain fatty acids

SCFAs are volatile fatty acids produced by intestinal bacteria with fewer than six carbons and are the most important metabolites in host-microbiota interactions. Bacteria express glycoside hydrolase which converts glycans into usable sugars. In the human genome no enzyme is capable of digesting glycans; indeed, many carbohydrates are digestible only by bacteria and produce SCFAs, the primary fuel for colonocytes. The most common SCFAs are acetic, propionic and butyric acid (in a molar ratio of 3:1:1), and they constitute 90%-95% of all SCFA present in the human colon.

In addition to being the main source of energy for the colonocytes, butyric acid is involved in maintaining the intestinal mucosa health state. Numerous effects have been highlighted both at the intestinal and extraintestinal level. Butyrate has been shown to inhibit inflammation, promote colonic healing in colitis (61) and reduce carcinogenesis (62) with mechanisms that include stimulation of apoptosis (63). Interestingly, acetic acid appears to play a central role in appetite suppression; in mice it has been demonstrated that acetic acid produced during colonic fermentation crosses blood-brain barrier and act through central hypothalamic mechanisms (64).

As previously stated, SCFAs may be used as energy source by the colonocytes, which can oxidize fatty acids to carbon dioxide and ketone bodies. Leftover SCFAs reach the liver through the portal circle where acetate is used as a precursor for the synthesis of cholesterol and long-chain fatty acids (65). Layden and collaborators (66) demonstrated that obese women who follow a Western diet rich in sugars, fatty acids, refined carbohydrates and low in fiber, have reduced levels of cholic acetate, which are negatively associated with visceral fat and fasting insulin levels. Propionic acid is the second most abundant SCFA and is largely taken up by the liver; it has a potential role in the reduction of lipogenesis, in the inhibition of cholesterol synthesis, in the increase of the sense of satiety, and has anti-inflammatory properties (67).

### 4.3 Microbiota and obesity

Many experimental and clinical studies have highlighted the complex role played by the intestinal microorganisms and their influence on multiple functions including the regulation of the neuro-immuno-endocrine system. As demonstrated by Buffington et al. (68), a change in the “core microbiome’’ can lead to the onset of obesity, as observed in twin pairs with reduced bacterial diversity and an enrichment of obesity-associated genes, 75% of which belong to Actinobacteria.

Other studies conducted in 2004 by the group of Gordon (57) and collaborators demonstrated a potential relationship between the intestinal microbiome and the development of an obese phenotype. An abundance of Firmicutes and a relative decrease of Bacteroidetes were found in the microbiota of obese mice. Colonization of adult germ-free mice using strains of bacteria taken from the distal intestine of conventional adult mice determined a dramatic increase in body fat within 10-14 days, despite a reduction in food consumption. The cause of these changes can be attributed to numerous mechanisms including the microbial fermentation of some food polysaccharides indigestible for the host, the consequent intestinal absorption of monosaccharides and SCFAs and their conversion in the liver into more complex lipids. Backed and collaborators have also demonstrated how germ-free mice are resistant to the typical Western diet rich in fats and sugars (69). Other studies using genetically obese mice (*ob/ob*) or Zucker obese rats (fa/fa) revealed differences in their “metabotypes” attributable in part to the presence of Bacteroidetes, Firmicutes, Actinobacteria in different proportions (70). Of note, faecal microbiota transplantation (FMT) from obese mice into lean germ-free recipient mice modifies body weight (71), as germ-free mice that received faecal microbiota of obese mice increased their body weight, whereas mice receiving a faecal microbiota from lean mice remained lean. The study also demonstrated that genes coding for enzymes involved in the degradation of food polysaccharides were enriched with a consequent increase in energy extraction. A study conducted on volunteers subjected to a body weight reduction program by using a low-calorie diet, demonstrated that the weight loss of obese individuals with a BMI>30 was accompanied by a significant increased number of Bacteroidetes, from 3% to 15%, which contributes to a better intestinal energy extraction (72). However, based on many studies on obese subjects, the relationship between Bacteroidetes and Firmicutes remains debated as differences in genotype and lifestyle are unresolved contributing factors. A recent review on the role of the gut microbiota in obesity analyzed 60 studies reporting that the phylum Proteobacteria is most frequently associated with obesity (73). Many Proteobacteria species are proinflammatory and have been associated with chronic inflammatory conditions such as Crohn’s disease and ulcerative colitis (74).

The complex interactions between environmental, genetic and behavioral factors are actually responsible for the etiology of obesity and its metabolic complications, including low-grade inflammation, hyperlipidemia, hypertension and diabetes. The metabolic activities of the gut microbiota facilitate the extraction of calories from ingested foods and help store calories in host adipose tissue for later use, providing energy and nutrients for microbial proliferation and growth. In turn, differences in calorie extraction may be due to the different compositions of the intestinal microbiota. Gut microorganisms favour fat storage in adipocytes through the inhibition of Fasting Induced Adipocyte Factor (FIAF), an inhibitor of lipoprotein lipase (LPL), consequently causing an increase in LPL activity, and thus promoting increased fatty acid uptake and the accumulation of triglycerides in adipocytes (69). This phenomenon occurs exclusively at the level of the intestinal epithelium, and not at the level of other areas, such as the liver, which continue to synthesize FIAF.

## 5 Intestinal microbiota interactions with the *endocannabinoidome*

There is no doubt that the microbiota affects gut physiology and its role in the gut brain-axis has been convincingly established (75). Many physiological roles of intestinal microorganisms are associated to the regulation of the intestinal endocannabinoid tone [the so called endocannabinoidome (49) as extensively described in recent exhaustive reviews (76–78). There is increasing evidence that both selected intestinal microorganisms and bioactive lipids covary in pathological conditions such as obesity, type 2 diabetes and inflammation (76). eCB and associated bioactive lipids are now considered to be putative ‘gate-keepers’ that contribute to securing the intestinal barrier and to reducing inflammation.

### 5.1 Endocannabinoids and intestinal microbiota regulate gut homeostasis

In this paragraph we report a few significative examples of the crosstalk between intestinal microbiota and eCB, whereas the following paragraph will be dedicated to the crosstalk between the paracannabinoids and intestinal microorganisms.

The gut microbiota and the eCB system are fundamental modulators of energy homeostasis and obesity, which is characterized by massive expansion of adipose tissue and is associated with inflammation (79). Obesity is also characterized by a relevant increase of endocannabinoids levels in both plasma and adipose tissue, decreased expression of FAAH, and altered expression of cannabinoid receptor 1 (CB1) (80). Whether the upregulated peripheral eCB system offers a protecting mechanism in obesity, remains to be established. The eCB system also regulates the intestinal barrier function: in obese *ob/ob* mice with metabolic endotoxemia and disturbed intestinal barrier, blocking the CB1 receptor, reduced food intake, markedly reduced intestinal permeability and plasma LPS levels (81). An interesting hypothesis of these authors holds that the eCB system links the development of intestinal permeability to higher LPS plasma levels associated with obesity. The same authors reported that in obese mice fed with prebiotics, CB1 receptor expression as well as anandamide contents in the colon were normalized, whereas FAAH expression was increased (81). LPS controls eCB synthesis both *in vivo* and *in vitro* through mechanisms that depend on LPS receptor signaling. It was suggested that LPS acts as a master switch that controls adipose tissue metabolism by blocking cannabinoid-mediated adipogenesis (81). Kuipers and collaborators (2019) demonstrated in mice that a High Fat Diet (HFD) rapidly activates the adipose tissue increasing endocannabinoids synthesis with aggravation of HFD-induced obesity. Elevated systemic LPS levels predispose to increased gut permeability by reduced expression of tight junction proteins which predisposes to LPS translocation resulting in endotoxemia and relative high level of pro-inflammatory cytokines (82–84).

The intestinal microbiota, therefore, determines the physiology of adipose tissue through the regulatory loops of the LPS-eCB system and could have critical functions in adipose tissue plasticity during obesity. The microbiota also modulates intestinal eCB tone; as an example, Rousseaux et al. (85) showed that the expression of intestinal epithelial CB_2_ receptor increases when mice or rats are orally administered the bacterium *Lactobacillus acidophilus*. More recently it was found that the bacterium *Akkermansia muciniphila* is a prominent regulator of the gut eCB tone, gut permeability, and secretion of gut peptides (86); see also (78) for a comprehensive review. The role of *A. muciniphila* in pathological conditions has also been investigated. For instance, *Akkermansia* spp. has been inversely related to the severity of irritable bowel disease, appendicitis and obesity, suggesting a protective or anti-inflammatory activity (reviewed by (87).

### 5.2 Paracannabinoids and intestinal microbiota regulate gut homeostasis

Much less is known about the interplay between the PEA, OEA and other biogenic lipids and the microbiota in the context of intestinal homeostasis. As an example, *A. muciniphila* regulates intestine levels of various fatty acid amides (FAA), among which 2-oleoyl glycerol, (a lipid mediator associated with protection against inflammation, as well as gut permeability), and the secretion of glucagon-like-peptide 1 (GLP-1) (86), a gut peptide with broad pharmacological potentials [see also (78) for a comprehensive review]. Recently some studies (88) questioned the beneficial effects of *A. muciniphila*, as plasma analyses of obese and overweight subjects treated for three months with daily ingestion of either alive or pasteurized *A. muciniphila* were not linked to an overall modification of the endocannabinoidome.

As mentioned in the previous paragraph, diet composition has a great impact on microbiota profile. It was recently shown that switching from Western diet to isocaloric Mediterranean diet decreases plasma levels of the FAA 2-arachidonoyl glycerol (2-AG), increases plasma PEA and OEA levels, and increases faecal *A. muciniphila* abundance (89, 90). These observations have clinical translational values, as the abundance of *A. muciniphila* is decreased in obese and type 2 diabetic mice, and the presence of this bacterium inversely correlates with body weight in both humans and rodents. A clinical trial conducted in obese people showed that the supplementation with OEA reduced body weight, energy intake and fat mass along with the significant increase in the abundance of *A. muciniphila* (91). Overall, these observations suggest a direct link between gut microbiota and intestinal endocannabinoidome which may constitute one of the pathways involved in the crosstalk between gut microbes and enteroendocrine host cells.

We recently described a previously unexplored modulatory effect of OEA on the intestinal microbiota profile, as well as on intestinal immune responses (52). OEA administered sub-chronically to mice fed a normal chow pellet diet changed the faecal microbiota composition, shifting the Firmicutes: Bacteroidetes ratio in favour of Bacteroidetes mostly of the *Bacteroides* genus. Further analysis indicated that the predominant *Bacteroides* in the microbiota of OEA-treated mice were attributable to *B. acidifaciens.* On the other hand, OEA treatment reduced significantly Firmicutes, especially the genus *Lactobacillus*, especially *L. reuteri* and *L. gasseri.* Prediction analysis of how the observed differences in microbiota profiles reflected enriched functional pathways in OEA-treated mice, showed, among others, enrichment of metabolic pathways associated with amino acids metabolism, (e.g., tryptophan and phenylalanine metabolism), lipoic acid metabolism, glycan and glycosaminoglycan degradation and reduced biosynthesis of unsaturated fatty acids. Overall, the consequences of OEA on the microbiota profile are comparable to those afforded by a fibre-rich diet which promotes the survival of saccharolytic bacteria, such as Bacteroides, which use glycans as energy sources. Therefore, it appears that the homeostatic and metabolic effects of OEA may change the intestinal environment and the ecological fitness of bacterial community.

OEA has also profound effects on the polarization of T_H_ lymphocytes in the Peyer’s patches, which are considered the immune sensors of the intestine, towards an anti-inflammatory profile (52). The observation that OEA changes the microbiota profile concomitantly with Peyer’s patches environment suggests a double, possibly related, effect of OEA in the intestine that may be exploited to counteract obesity-induced, local and systemic inflammation. In this regard, there are other controversial studies regarding the direct interaction between intestinal microbiota and OEA levels. In mice, microbiota disruption with a cocktail of antibiotics did not modify OEA levels in the intestine (92), whereas a more recent study showed that OEA levels in mice caecum were significantly decreased after antibiotic treatment (93), suggesting that the intestinal microbiota is responsible in part for the production of OEA.

In a very elegant study using GF mice, Manca et al. (94) demonstrated relevant changes in eCBome signalling that are partially reversed by faecal microbiota transplant (FMT). GF mice were characterized by global changes in eCBome gene expression, and colonization by intestinal microbiota following FMT partially reversed this effect. Hence, these results provide a cause-effect relationship between the presence or absence of gut microbiota and endocannabinoidome signalling. As mentioned previously, OEA controls the secretion and efficacy of GLP-1, suggesting a synergistic actions of this FAA with intestinal microorganisms in the regulation of several homeostatic functions, as GLP-1 has numerous metabolic actions among which decrease gastric emptying, inhibition of food intake, glucose-dependent stimulation of insulin secretion (95).

A recent study conducted in normal subject exposed to 6-week exercise intervention and a validation cohort (96) revealed that at baseline, eCB and paracannabinoid levels were associated with higher microbiome diversity, negatively associated with *Escherichia/Shigella* and *Collinsella*, whose increased levels are found in type 2 and gestational diabetes (97), and negatively associated with weight loss and insulin sensitivity (98). Levels of endo- and paracannabinoids were also associated with higher levels of the microbiota-produced SCFA butyrate, along with increases in the anti-inflammatory cytokine IL-10 and decreases in pro-inflammatory cytokines like IL-8 and TNFα (96). These data demonstrate that the anti-inflammatory effects of SCFAs are in part mediated by the *endocannabinoidome*, suggesting the existence of other pathways used by the gut microbiome for the modulation of the immune system.

The most compelling study that unequivocally causally links OEA to intestinal microbiota was recently published by the group of Christoph Thaiss (99). Their elegant paper shows that physical activity is not strictly regulated by the central nervous system, but is shaped by peripheral factors originating in the intestinal microbial community. The authors discovered that certain gut bacteria enriched in exerting mice contribute to the production of OEA that excites TRPV1^+^ sensory neurons. These send an exercise-induced afferent signal to the brain, indirectly elevating dopamine levels in the ventral striatum during exercise. The authors suggest that gut-derived interoceptive circuits are in part responsible for the rewarding mechanisms of exercise.

## 6 Conclusions

The intestinal microbiota is without any doubt one of the key elements contributing to the regulation of host health and it is tightly connected to the bioactive lipids belonging to both the endocannabinoidome systems. Indeed, the fact that both these systems coexist together with gut microorganisms and are maintained through evolution, points to a strict physiological relationship between them to ensure the regulation of dynamic process of the host metabolism.

We may suggest that in this scenario OEA acts as a *trait-d’union* between gut microbiota and dynamic physiological and homeostatic processes. In this article we reviewed data suggesting that the malfunctioning of the crosstalk between intestinal microorganisms and the *endocannabinoidome* is responsible for intestinal dysfunctions, enteropathies, and a variety of disorders such as obesity and associated chronic inflammatory state. OEA produced in the gastrointestinal tract is a major component of the gut-brain axis contributing to complex communication between the periphery and the central nervous system, hence linking cognitive and emotional brain center with peripheral functions (100). Many pieces of the puzzle are still missing to fully explain the communication between the host and its gut microbiome. Although environmental factors have a markedly stronger effect on microbiota composition, the host genetics as well may influence the microbiota profile (101), which adds further complication to the whole scenario. Nonetheless, the identification of new signalling pathways connecting the intestinal microbiota with the host physiology is very fascinating and holds great promise for the development of novel therapeutic strategies to cure metabolic, inflammatory, and cognitive disorders, which may represent valuable and safer alternatives to current treatments.

## Author contributions

All authors contributed to the writing and agreed on the final version of the manuscript.
